# Computational Functional Genomics-Based AmpliSeq™ Panel for Next-Generation Sequencing of Key Genes of Pain

**DOI:** 10.3390/ijms22020878

**Published:** 2021-01-16

**Authors:** Dario Kringel, Sebastian Malkusch, Eija Kalso, Jörn Lötsch

**Affiliations:** 1 Institute of Clinical Pharmacology, Goethe-University, Theodor-Stern-Kai 7, 60590 Frankfurt am Main, Germany; kringel@med.uni-frankfurt.de (D.K.); malkusch@med.uni-frankfurt.de (S.M.); 2 Department of Anaesthesiology, Intensive Care and Pain Medicine, University of Helsinki and Helsinki University Hospital, P.O. Box 440, 00029 HUS Helsinki, Finland; eija.kalso@helsinki.fi; 3 Fraunhofer Institute for Translational Medicine and Pharmacology ITMP, Theodor-Stern-Kai 7, 60596 Frankfurt am Main, Germany

**Keywords:** next generation sequencing, human genomics, pain genetics, pharmacogenomics, computational functional genomics, data science, knowledge discovery

## Abstract

The genetic background of pain is becoming increasingly well understood, which opens up possibilities for predicting the individual risk of persistent pain and the use of tailored therapies adapted to the variant pattern of the patient’s pain-relevant genes. The individual variant pattern of pain-relevant genes is accessible via next-generation sequencing, although the analysis of all “pain genes” would be expensive. Here, we report on the development of a cost-effective next generation sequencing-based pain-genotyping assay comprising the development of a customized AmpliSeq™ panel and bioinformatics approaches that condensate the genetic information of pain by identifying the most representative genes. The panel includes 29 key genes that have been shown to cover 70% of the biological functions exerted by a list of 540 so-called “pain genes” derived from transgenic mice experiments. These were supplemented by 43 additional genes that had been independently proposed as relevant for persistent pain. The functional genomics covered by the resulting 72 genes is particularly represented by mitogen-activated protein kinase of extracellular signal-regulated kinase and cytokine production and secretion. The present genotyping assay was established in 61 subjects of Caucasian ethnicity and investigates the functional role of the selected genes in the context of the known genetic architecture of pain without seeking functional associations for pain. The assay identified a total of 691 genetic variants, of which many have reports for a clinical relevance for pain or in another context. The assay is applicable for small to large-scale experimental setups at contemporary genotyping costs.

## 1. Introduction

A genetic background of pain plays a role in rare hereditary extreme phenotypes that cause either pain insensitivity [1] or paroxysmal pain disorders [2], in the perception of acute pain [3], in the risk of pain persistence after a triggering event [4], or in the response to pharmacological [5] or non-pharmacological [6] pain treatments. The involvement of 540 “pain genes” in pain is supported by robust evidence [7,8], and further suggestions have been communicated [9,10]. With predominantly small effects exerted by common genetic variants [11], a breakthrough in the genetic profiling of individual risks, as occasionally expected [12], has not yet really been achieved [13]. Instead, this seems to be linked to a complex pattern of functional genetic variants [14], which is being discovered in an evolutionary rather than revolutionary way, which is supported by technical advances over the last decade [15] that allow to establish genotype versus phenotype associations for thousands of genetic variants in a still manageable small number of patients [14,16].

As a basis for the association of human genotypes with pain and the risk for its persistence, we propose a set of 29 genes as key players among the currently known pain-relevant genes [17]. Specifically, the functional genomics-based architecture of pain has been presented as a polyhierarchy of biological processes [8] based on the organization of the Gene Ontology knowledge base that captures the current knowledge about the biological roles of all genes and their respective products [18,19]. With the 29 genes, a respective representation created with 540 pain-relevant genes could be reconstructed by 70%, based on a bioinformatics analysis of the Gene Ontology knowledge base [17].

**Figure 1:**
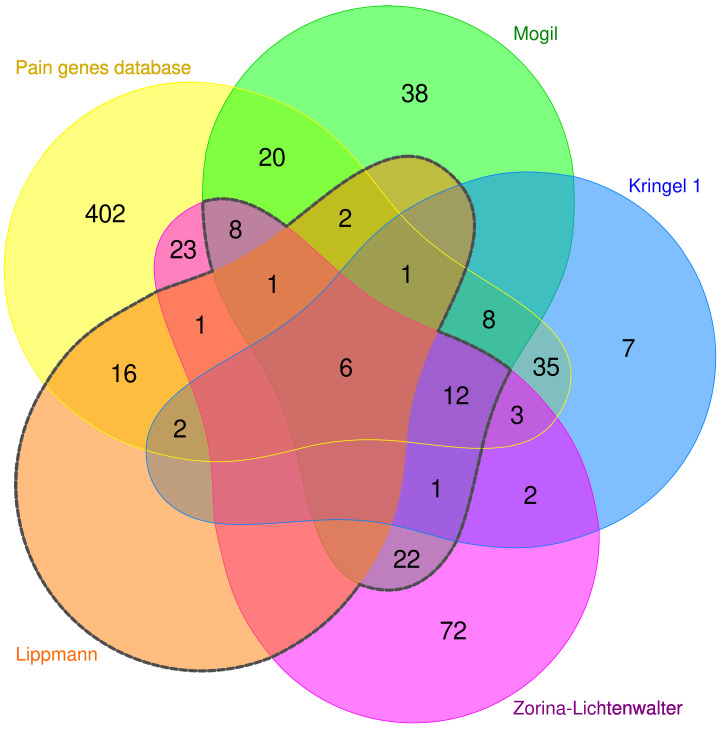
Assembly of the pain-relevant gene set forming the proposed NGS panel from various sources of evidence. The Venn diagram [90] visualizes the overlaps between the 29 key genes in the functional genomic representation of pain (“Lippmann” [17]) (subset 1 of the present NGS panel) and the two independent alternative proposals (“Mogil” [9] and “Zorina-Lichtenwalter” [10]) included as subset 2. The colors of the areas correspond to the colors of the adjacent names of the respective gene set. In addition, a set of d = 540 genes is indicated which have been empirically identified as relevant to pain and are either listed in the PainGenes database (http://www.jbldesign.com/jmogil/enter.html [7]) or were recognized as causing human hereditary diseases associated with extreme pain phenotypes, regulated in chronic pain in at least three studies including human association studies, or being targets of novel analgesics [91]. In addition, a further set of genes is included that belong to an NGS panel in an earlier approach to human genes relevant for the persistence of pain (”Kringel 1” [20]) The black dashed line surrounds the genes of the present NGS panel. The figure has been created using the R software package (version 4.0.2 for Linux; http://CRAN.R-project.org/ [92]) and the library “venn” (https://cran.r-project.org/package=venn [93]).

The present report describes the development of a genotyping assay for these 29 genes, with extension by further genes based on proposed importance for persistent pain to take full advantage of the technical specifications of the AmpliSeq^TM^ gene sequencing library technique (Figure 1), resulting in a set of d = 72 genes (Table 1) continuing the research path of functional genomics of pain that has been pursued in previous reports [7,9,10,20]. Here, (i) the assembly of the present set of genes is reported along with (ii) a computational analysis of its functional genomics and (iii) its establishment in a subset of samples from a cohort of patients undergoing breast cancer surgery [21], together with (iv) an evidence-based analysis of known functional implications of the variants identified in these samples, although without aiming for a functional association in the present cohort.

## 2. Results

### 2.1. Participants and Descriptive Data

The NGS assay of the proposed set of 72 human genes relevant for persistent pain, was established in 61 genomic DNA samples available from a cohort of patients after breast cancer surgery [94] and including 55 subjects without pain and six patients with persistent pain, which corresponded to the ratio of persistent pain versus no pain in the entire cohort in order to resemble a random sample of subjects in terms of pain as much as possible.

### 2.2. Main Results

**Figure 2:**
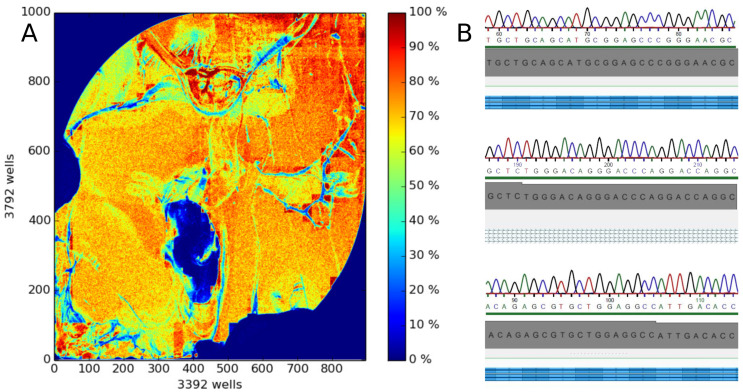
Technical detail of assay establishment and validation. (**A**): Pseudo-color image of the Ion 318TM v2 Chip plate showing percent loading across the physical surface. This sequencing run had a 76% loading, which ensures a high Ion Sphere Particles (ISP) density. Every 318 chip contains 11 million wells and the color scale on the right side conduces as a loading indicator. Deep red coloration stays for a 100% loading, which means that every well in this area contains an ISP (templated and non-templated) whereas deep blue coloration implies that the wells in this area are empty. (**B**): Alignment of segments of the ion torrent sequence of the *COMT* gene as Golden Helix Genome Browse^®^ readouts versus the same sequence according to an externally predicted Sanger electropherogram. The figure has been created using the original outputs of the Ion PGM System (Life Technologies, Darmstadt, Germany) and the Golden Helix Genome Browse^®^ software (Version 2.0.4, Golden Helix, Bozeman, MT, USA).

As applied previously [95], only exons including 25 bases of padding around all targeted coding regions for which the realized read-depths for each nucleotide was higher than 20 were contemplated as successfully analyzed. With this acceptance criterion, the whole or almost whole coverage of the relevant sequences was obtained. The NGS sequencing process of the whole patient cohort required seven separate runs, each with samples of *n* = 9 or *n* = 10 patients. Coverage statistics were analogous between all runs and matched the scope of accepted quality levels [20,21,94]. A median of 4.55 × 10^6^ reads per run was produced. The mean depth was close to 200 reads, the mean read length of called bases resulted in 215 bases and average chip loading was 67% (Figure 2). To establish a sequencing output with a high density of ISPs on a sequencing chip, the chip loading value should exceed 60% (Life Technologies, Carlsbad, CA, USA). The generated results of all NGS runs matched with the results obtained with Sanger sequencing of random samples, meaning the accordance of nucleotide sequences between next generation sequencing and Sanger sequencing was 100% in all validated samples.

**Figure 3:**
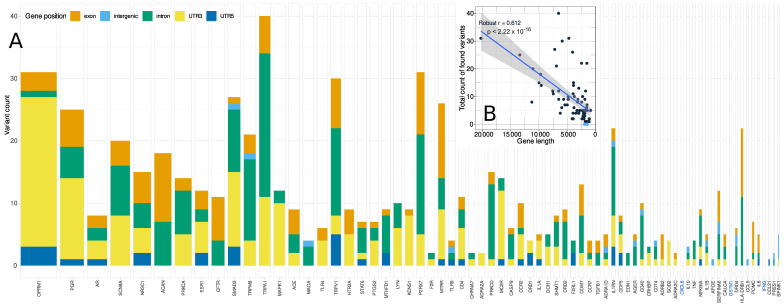
Number and localization of variants identified using the present AmpliSeq^TM^ panel, in relation to the read DNA length per gene. (**A**) Stacked bar plot representing the number of genetic variants per gene included in the assay, categorized for the gene locations. The horizontal size of the cells is proportional to the number of nucleotides assayed in the respective gene. The genes are ordered for descending read length. Variants were not found in three genes (*IFNG*, *GSTM1* and *CXCL8*; indicated in blue gene symbols at the *x*-axis), which are among the shortest genes. (**B**) Scatterplot of the total number of variants versus the number of nucleotides read for the respective gene in the present assay. A robust regression line with 95% confidence interval is overlaid on the dot plot. The genes where no variants had been detected are indicated as blue dots. Please note the decreasing order of gene length on the abscissa to match the main panel. The figure has been created using the R software package (version 4.0.2 for Linux, city, http://CRAN.R-project.org/ [92]) and the R libraries “ggplot2” (https://cran.r-project.org/package=ggplot2 [96]). UTR: untranslated region.

Following elimination of nucleotides agreeing with the standard human genome sequence GRCh37 g1k (dated February 2009), the result of the NGS consisted of a vector of nucleotide information about the d = 69 genes for each individual DNA sample. This vector had a length equaling the set union of the number of chromosomal positions in which a non-reference nucleotide had been found in any probe of the actual cohort. Specifically, a total of 691 genetic variants were found, of which 161 were exonic, 22 intergenic, 255 intronic, and 215 variants were located in the 3′-UTR and 38 variants in the 5′-UTR (Figure 3). Three genes (*IFNG*, *GSTM1,* and *CXCL8*) were not represented in the final set of genetic variants. Panel design and assay quality parameters were re-examined with positive results. The read gene length provided an explanation for the absence of variants (Figure 3). That is, the three genes were among the shortest genes in the available panels. In fact, the number of variants detected was significantly correlated with the total number of nucleotides read (total number of variants: robust correlation coefficient: 0.612, *p* = 1.116 × 10^−8^, exonic variants only: robust correlation coefficient: 0.398, *p* = 0.00054). The number of nucleotides read per gene also matched well with the gene length queried from the database “org.Hs.eg.db” (robust correlation coefficient: 0.8633, *p* < 2.22 × 10^−16^.

### 2.3. Other Analyses

**Figure 4:**
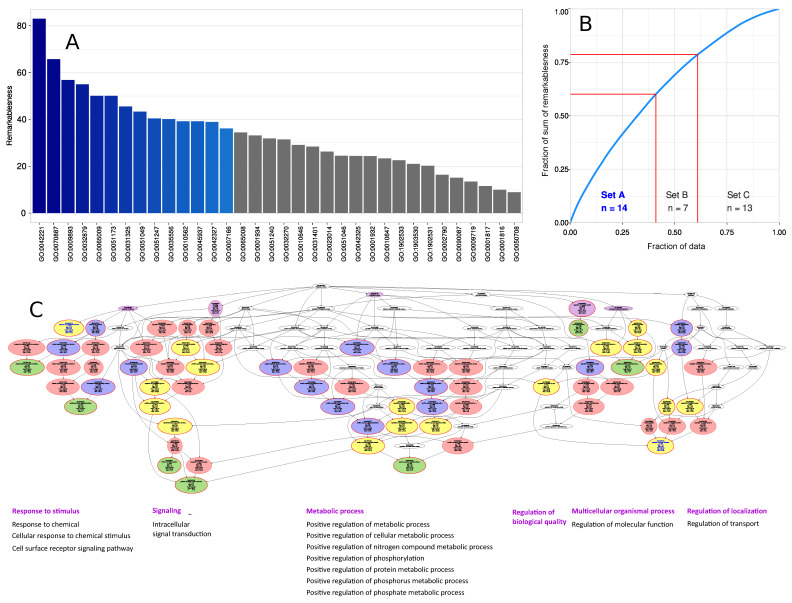
Computational functional genomics perspective on the biological processes in which the genes analyzed with the proposed NGS panel are involved. The figure displays the results of an overrepresentation analysis (ORA; *p*-value threshold, *t_p_* = 5 × 10^−15^ and Bonferroni α correction) of the 72 genes included in the present NGS panel (Table 1). (**A**) Bar plot of the gene relevance in the functional genomics representation of the present gene set. As a basis for the selection of the most relevant terms to describe the directed acyclic graph (DAG [97]) representing the polyhierarchical structure of the Gene Ontology database, i.e., the terms that can serve as headlines for each branch of the DAG, the remarkableness measure was previously introduced [98]. The bar plot shows the relevance of GO terms in decreasing order of the remarkableness measure. The blue bars indicate the most relevant terms selected by an item categorization technique, implemented as a computed ABC analysis [99]. (**B**) The ABC plot (blue line) shows the cumulative distribution function of the remarkableness measure with the limits between sets A, B and C indicate as red lines. The results show that 14 GO terms belonged to ABC set “A” and were therefore considered as most relevant to the DAG. (**C**) Top-down representation of the annotations (GO terms) representing a systems biology perspective of the biological processes modulated by the set of 72 genes included in the present NGS panel. Each ellipse represents a GO term. The graphical representation follows the standard of the GO knowledge base, where GO terms are related to each other by “is-a”, “part-of”, “has-a” and “regulates” relationships forming a branching polyhierarchy organized in a directed acyclic graph (DAG [97]). The color coding is as follows: No color: GO terms that are important for the DAG’s structure but do not have a significant *p*-value in Fisher’s exact tests. Red: Significantly overrepresented nodes. Green: Terms at the end (detail) of a branch of the DAG. In addition, the node’s text will be colored in blue to indicate that this node is a detail. Yellow: Significant nodes with highest remarkableness in each path from a detail to the root, i.e., the so-called “headlines”. The margins indicate over by its red color. Violet: Functional areas, i.e., terms selected to describe the parts below them in the DAG most concisely. The figure has been created using the R software package (version 4.0.2 for Linux; http://CRAN.R-project.org/ [92]) and the R libraries “ABCanalysis” (http://cran.r-project.org/package=ABCanalysis [99]), “ggplot2” (https://cran.r-project.org/package=ggplot2 [96]) and “dbtORA” (https://github.com/IME-TMP-FFM/dbtORA [100]).

The d = 29 genes have been shown to cover 70% of the DAG emerging from 540 pain genes, I.e., can be regarded to represent pain completely to this extent [17]. However, as the present panel had been filled with further genes, the present analyses aimed to functionally characterize the set of d = 72 genes. This was approached by computational querying of the knowledge about the function of human genes recorded in the knowledge base of gene ontology (GO). Over-representation analysis (ORA) identified 70 GO terms as significantly associated with the set of 72 genes, more often than randomly expected, at the selected *p*-value threshold of 5 × 10^−15^ with correction for multiple tests according to Bonferroni. Computed ABC analysis of the remarkableness of the GO terms qualifying as headlines to describe significant branches of the obtained polyhierarchy categorized d = 14 terms into ABC set “A” indicating the most important items (Figure 4). Further reduction of the number of GO terms by subsumption of adjacent branches of the polyhierarchy to the next suitable unifying term upwards the hierarchy led to six functional areas covered by the 72 genes.

**Figure 5:**
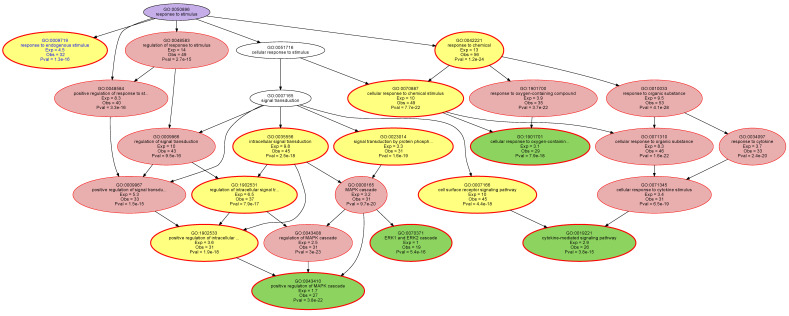
Detail of the directed acyclic graph (DAG [97]) shown in Figure 4, displaying the polyhierarchical structure of the Gene Ontology database (“point of view”) below the GO term “response to stimulus” (GO:0050896). This was one of the major biological processes identified by a functional genomics analysis aiming at characteristics of pain and defined as “Any process that results in a change in state or activity of a cell or an organism (in terms of movement, secretion, enzyme production, gene expression, etc.) as a result of a stimulus. The process begins with detection of the stimulus and ends with a change in state or activity or the cell or organism” [19]. The color coding is as follows: No color: GO terms that are important for the DAG’s structure but do not have a significant *p*-value in Fisher’s exact tests. Red: Significantly overrepresented nodes. Green: Terms at the end (detail) of a branch of the DAG. In addition, the node’s text will be colored in blue to indicate that this node is a detail. Yellow: Significant nodes with highest remarkableness in each path from a detail to the root, i.e., the so-called “headlines”. The margins indicate over by its red color. Violet: Functional areas, i.e., terms selected to describe the parts below them in the DAG most concisely. The figure has been created using the R software package (version 4.0.2 for Linux; http://CRAN.R-project.org/ [92]) and the library “dbtORA” (https://github.com/IME-TMP-FFM/dbtORA [100]).

These areas included: (i) the “regulation of localization” (GO:0032879), which mainly concerned the regulation of protein secretion (GO:0050708); (ii) “response to a stimulus” (GO:0050896) (Figure 5), especially to a chemical stimulus (GO: 0042221) and converged to “cytokine-mediated signaling (GO:0019221) and the MAPK cascade (GO:0043410); (iii) the “metabolic process” (GO: 0008152), which also converged to the MAPK cascade, the ERK1 and ERK2 cascade (GO: 007031), and the protein kinases (GO:0045860); (iv) the “multicellular organism process” (GO:0032501), which mainly involves cytokine production (GO: 0001819), (v) “signaling” (GO:0023052), which in turn converges to the MAPK cascade, and (vi) finally, the non-specific “regulation of biological quality” (GO:0065008), which is considered to be relevant in the maintenance of homeostasis. Taken together, the set of *n* = 72 genes was functionally mainly involved in the mitogen-activated protein kinase of extracellular signal-regulated kinase, which, in interaction with cytokine production and secretion, indicated the control of immune and inflammatory processes in pain.

## 3. Discussion

The primary subset of the present panel of pain-relevant genes represents key genes of pain that had emerged in a computational functional genomics-based analysis that considered the position of biological processes in which these genes were involved in the polyhierarchical presentation of pain [17]. In a previous analysis of the functional genomics of pain [8], the biological functions characterizing pain had been identified to comprise 12 different components. Specifically, main functional areas were “behavior”, “response to wounding” and “response to organic substance”, which are sub-terms of the GO term “response to stimulus”. In addition, ion homeostasis and transport, the synaptic transmission of nociceptive input and intracellular signal transduction including the G-protein coupled receptor- signaling pathway as well as anatomical structure development and regulation of (multicellular) system processes completed the full functional picture of pain. In a later analysis [17], the present subset 1 of *n* = 29 best-scoring genes was found to identify the GO terms forming the complete polyhierarchy with precision and recall of more than 70%. Thus, the present subset 1 includes genes which best reflect the functional biology of pain. For comparison, when using a random sample of *n* = 29 genes from the 540 pain-relevant genes, the average recall of the GO terms of the pain-DAG was only 1.77% [17]. The relevance for pain in general was also supported by the observation that for the currently 29 genes a significantly higher hit rate of drug targets was achieved than for a random sample of 29 genes among the 540 pain genes [17]. Hence, several lines of evidence provide support that subset 1 can be considered as a pain-relevant selection of genes.

It was technically possible to add more genes without increasing the analytical cost, and this option was chosen by adding subset 2, which uses previous and independent efforts to select pain-relevant genes [9,10]. This shifted the functional genomics of pain covered by the resent NGS Panel, resulting in the need for a new analysis, which led to an emphasis on immune and inflammatory processes in the functional genomics of pain covered by the current NGS panel, particularly represented by the mitogen-activated protein kinase of extracellular signal-regulated kinase and cytokine production and secretion.

Thus, the present panel provides a key set of pain genes that has been derived from a computer-aided functional genomics analysis [17] of 540 genes of the PainGenes database [7]. Although this covers 70% of the biological processes in which the 540 genes are involved, it is not an exhaustive set of genes of interest for pain. The genes in the PainGenes database were included based on studies in transgenic mice, with the condition that at least one statistically significant difference was reported between the mutated mice and their concurrently tested wild-type controls. However, alternative approaches, including by the authors of the PainGenes database [9], used different criteria, such as reported associations with clinical pain, resulting in additional gene sets that were suggested to be pain relevant. A selection of these genes was included as subset 2 in the present panel, and in addition, additional genes from these proposals were included in an earlier, similarly designed NGS panel [20,95,101]. For example, the *COMT* genes were added, which were extensively studied in connection with pain modulation [102], but were not included in the important subset 1 of the present panel, but were members of subset 2.

### 3.1. Discussion of Main Results

#### 3.1.1. Technical Considerations

Since 2008, when sequencing switched from Sanger-based to NGS technologies, the cost per raw megabase has been significantly below the expectations predicted by the reciprocal of Moore’s law, where the latter is an empirical observation from computer hardware engineering and describes technological developments that are widely regarded as successful (for data, see https://www.genome.gov/about-genomics/fact-sheets/DNA-Sequencing-Costs-Data). The panel presented here, fits well into the current costs. Our sequencing project with the Ion Torrent^TM^ platform using the personal genome machine with 318^TM^ chips cost approx. € 630 per sequencing run while the cost per raw megabase of the 72 genes in 56 DNA samples required approximately € 0.20 per Mb. While the sequencing cost per run for the Torrent^TM^ and Ilumina MiSeq are comparable, the investment costs for the MiSeq sequencer are higher, but this machine has a higher throughput, which reflects in a slightly reduced sequencing cost per megabase [103].

#### 3.1.2. Functional Involvement of Genes in Biological Processes

The selection of genes relied on empirical evidence of their involvement in pain. For subset #1 (d = 29). This had been shown for all of the genes in the original paper [17]. This subset includes d = 29 genes identified using a computational functional genomics-based approach in which the gene sets are reduced to the most relevant items based on the importance of the gene within the polyhierarchy of biological processes characterizing pain. Subset #2 resulted from two proposed sets of human genes involved in modulating the risk or clinical course of persistent pain “Mogil” [9] and “Zorina-Lichtenwalter” [10]. The chosen set of genes for subset ‘2 includes the intersection from both alternative proposals aiming at similar phenotypes. However, when analyzing these alternatives for mutual agreement, an overlap of *n* = 50 could be observed. Combining all proposals into a large panel was not an option due to the technical limitations of the IonTorrent^TM^ restricting the panel size to 500 kb (pipeline version 5.6.2, Carlsbad, CA, USA).

Both subsets comprised genes associated with the mesolimbic dopaminergic system, i.e., *DRD1*, *DRD2*, *DRD3*, *DRD4* which code for dopamine receptors which play an important role in pain modulation, suggesting that dopamine can modulate pain signals by acting at both presynaptic and postsynaptic targets [104]. Further genes were involved in cytokine production (*CCL21* *CCL5* *CCR2*, *CCR7*) and there is significant evidence showing that certain cytokines are involved in not only the initiation but also the persistence of pathologic pain by directly activating nociceptive sensory neurons [105]. Another main focal point were genes associated with immune regulatory processes including genes coding for interleukins (*IL1A*, *IL6*, *IL10*, *IL1B*, *IL1RN*) [106,107,108,109] and the histocompatibility complex related gene *HLA-DRB1* [110], which has been shown to be involved in immunological mechanisms of pain [111]. This is also supported by published evidence for the further genes in this list, such as TNF [112], GCH1 [113], and P2RX7 [114]. The view of pain and its development towards persistence as a trait resulting from alterations in the immune system is a concept that is biologically highly plausible and agrees with other lines of pain research and has been discussed more detailed in a previous work [115]. Another major process group included members of the transient receptor potential (TRP) family (TRPA1, TRPM8, TRPV1) that are expressed at nociceptors and which are well established players in the perception of pain [116]. This similarly applies to the Toll-like receptor genes (*TLR4* and *TLR9*)), which have been associated with the inflammatory consequences of glia activation (including microglia and astrocytes), sensory neurons, and other cell types which can influence nociceptive processing [47].

#### 3.1.3. Functional Involvement of Detected Variants

In the present study sample, a total of 691 genetic coding variants were found. Regardless of the sample preselection, 68 clinical associations, of which 29 have associated with various painful conditions (Table 2), could be queried for the observed variants from open access data sources. These comprise: (i) the Online Mendelian Inheritance in Man (OMIM^®^) database (https://www.omim.org), (ii) the NCBI gene index database, the GeneCards database (https://www.genecards.org) and the 1000 Genomes Browser (all accessed in October 2020). Although the present gene set has been assembled with a focus of a relevance to pain, many of its members have been implicated in pharmacogenetic modulations of drug effects (Table 3). Moreover, several of the genes in the present NGS panel have been chosen as targets of analgesics, approved or under current clinical development (data not shown). Functional polymorphisms that have been proven to influence gene functions are the most common candidate mutations in human that play a vital role in the genetic basis of certain diseases [111,117].

For example, a single nucleotide polymorphism determined as ER22/23EK (rs6189 and rs6190) is located in the exon 2 of the glucocorticoid receptor gene (*NR3C1*) and involves codons 22 and 23. This SNP is revered be responsible for relative resistance to glucocorticoids [111] and is associated with several effects like mood and anxiety disorders in patients with asthma [191] and a more aggressive disease course in multiple sclerosis [111]. Results of a study of the functional consequences of *P2RX7* polymorphisms in recombinant cells in vitro [132] suggested a correlation between gain-of -function and loss-of-function of *P2RX7* expression. It was further demonstrated that in patients with diabetic peripheral neuropathic pain (DPNP), the presence of the gain-of-function SNPs rs208294 (His155Tyr) is associated with higher pain intensity scores. Another meta-analysis addressed the role of *P2RX7* SNP (rs1718119) with the odds of Tuberculosis [192] and the findings indicate that this polymorphism could serve as a potential biological marker. Another recent study aimed to examine whether pharmacogenetics explains some of the variability in the response to fentanyl, which is an agonist of the μ-opioid receptor commonly used in the treatment of moderate-severe pain. Carriers of the C523A polymorphism (rs1042718) in the *ADRB2* gene were associated with increased response to fentanyl [111].

#### 3.1.4. Comparison to other Proposals of Pain-Relevant Gene Assay Panels

The present panel for sequencing genes associated with pain complements alternative proposals, including commercial offerings of “pain gene” sets that promise to provide ready-to-use assays for private testing or clinical association studies. For example, the company GX Sciences (Austin, TX, US) advertises a panel containing 30 single nucleotide polymorphisms in 28 different genes that comes with a do-it-yourself saliva swab test kit. All genes have a referenced relationship to chronic pain, but no further insight into the gene selection criteria is provided, nor is there any further information on the genotyping method performed (https://www.gxsciences.com). This panel and the currently proposed one have only 8.3% of their total genes in common, while 42.8% of the GXS panel are so-called pain genes, i.e., genes mainly from the PainGenes database (http://www.jbldesign.com/jmogil/enter.html [7], with some extensions [8] comprising targets of approved analgesics [91] and genes known to be causally involved in familiarity syndromes with either absent or paroxysmal exaggerated pain [1]. For comparison, 65.3% of the presently proposed panel are “pain genes”.

Another alternative panel for sequencing pain-related genes is provided by Live Technologies (Carlsbad, CA, USA), the manufacturer of the Ion Torrent^TM^ platform used for the present panel. It includes the complete exonic sequence of 64 pain-related genes. However, without further information on gene selection criteria (https://www.ampliseq.com). This set and the currently proposed set have only 11.5% of their total genes in common. However, 59.5% of this panel also belongs to the so-called “pain genes” mentioned above [8]. A third alternative is a cloud-based, publicly available knowledge base that enables virtual gene panels for human diseases and includes a virtual panel for chronic pain with 28 genes contributed by various departments, research groups, and consortia (https://panelapp.genomicsengland.co.uk). This set shares only 2% of the genes with the present panel, but 59.5% of its genes are also included in the “pain gene” set [8].

Thus, the present set of genes fits and complements other proposals. Taken above-mentioned proposals, the panel introduced in the present report and our previous panel [20] together, NGS sets for pain already cover 28.3% of the 540 genes included as references in the “pain-genes” set, which is based on the most stringent inclusion criteria by also requiring independent validations of a gene’s involvement in pain in knock-out models [7]. In contrast to the alternatives, the present set of pain-related genes is mainly based on a selection resulting from a computer-assisted functional genomic analysis [17]. According to a computational analysis of the functional involvement of the gene set [17], it covers > 70% of the genetic architecture of pain. This outperforms alternative proposals that purportedly single out pain-relevant genes but seem to lack a clear functional hypothesis. The apparent discrepancies between the different proposals, which also extend to the sets of pain-related genes used to complete the present panel, underscore that the genetic architecture of persistent pain is still incompletely understood and that several independent lines of research can be pursued, because combining all proposals into a large research panel is not yet an easily implemented option because of the technical limitations of NGS applications. The development of these panels is aimed at broadening the genetic perspective on pain. Indeed, although many candidate gene association studies have identified multiple genes relevant for pain phenotypes in the past decade, but roughly ten genes or gene complexes account for over half of the findings and several of these candidate gene associations have held up in replication [9]. 

### 3.2. Strengths and Limitations

The present AmpliSeq^TM^ panel complements earlier proposals on genes relevant for pain and especially persistent pain [7,9,10,20] and provides a validated assay suitable for high-throughput analyses to further evaluate genetic biomarkers for pain in this clinical setting. The biological roles of the included genes are clearly defined by the functional-genomics description based on the current acquired knowledge about higher-level organization of gene products into biological pathways [193], of which the gold-standard is the Gene Ontology (GO) knowledge base [19]. These include a set of genes that have been shown to be essential for pain in a bioinformatics approach [17], which is in the present report regarded as the primary subset of major importance. Among the limitations, firstly, the selection of test persons does not reflect a random sample of a population, but only includes women with breast cancer. An attempt was made to reduce bias towards or against persistent pain by maintaining the respective ratio observed in the original cohort [94]. However, this proportion may not be identical for different settings of persistent pain. Furthermore, the inclusion of only women may have distorted the frequency of the X chromosome variant observed in this analysis.

It is important to emphasize that the present report is limited to the details of assay development including the gene selection process. The separate report of the panel development provides the details of its establishment and validation, along with the computational genomic bases of the gene selection and the functional implications of the selected gene set in the context of previous proposals of important genes related to persistent pain. Hence, it can be considered as a separate scientific analysis that would exceed the necessary explanations provided within a genetic association report. In particular, the selection of the main subset of the present panel is based on a functional analysis and thus goes beyond the collection of genes discussed in the previous subsection, which seems to provide rather random collections based solely on mentions in the literature as pain-relevant genes.

In contrast, the genetic analyses for risk of persistent pain will be performed in a cohort of 70/70 women with persistent/non-persistent pain, extending a previous analysis [14]. After all, the immediate exploitation of the advanced technology which NGS provides over single variant analysis, which was still common a decade ago, is still easier to achieve in limited gene sets than in the whole genome due to technical limitations. In view of the high prevalence of chronic pain of about one fifth to one third of the European population [194,195], it is important to advance the discovery of genetic markers as quickly as possible.

## 4. Materials and Methods

### 4.1. Assembly of a Pain-Relevant Gene Set

The present NGS panel of pain relevant genes (Table 1) comprises two subsets derived (i) from a computational functional genomics bioinformatics approach to key players in the genetic architecture of pain [17] and (ii) further genes taken from independent proposals of published evidence-based genes relevant to (persistent) pain.

#### 4.1.1. Computational Functional-Genomics Based Key Genes for Pain

The focus in the selection of genes was on maintaining, as completely as possible, the functional genomics picture of pain with a reduced number of genes, as had been achieved by applying the computational functional genomics-based method of reducing disease-related gene sets to their key components [17]. Thus, subset 1 (Table 1) of the presently proposed NGS panel consisted of the 29 genes with which it had been possible to reproduce the biological processes in which the full set of 540 pain-relevant genes is involved by over 70% although they represented only 5% of the original genes.

#### 4.1.2. Published Evidence-Based Genes Relevant to Pain

In order to fully exploit the technical potential of the NGS panel, another 50 genes were added as subset 2 (Table 1). Specifically, these genes were selected based on intersections between two independently proposed alternative sets of human genes involved in modulating the risk or clinical course of pain and its persistence (Figure 1). The sets contained 127 genes [9] and 152 genes [10]. Their intersection included 50 genes. As subset 1 and subset 2 shared seven genes, the present NGS panel included 72 unique genes.

### 4.2. Establishment of the AmpliSeq^TM^ NGS Panel

#### 4.2.1. DNA Sample Acquisition

The present set of genes complements an earlier NGS panel [20], which was successfully applied to genotype versus phenotype associations in patients who had undergone breast cancer surgery [14]. The laboratory analyses were therefore performed on the same DNA samples that were used previously, but with a non-redundant technical implementation. The samples comprise a subset from a cohort of *n* = 1000 women with unilateral non-metastatic breast cancer, which has already been reported in connection with the development of persistent pain after surgery [21]. All subjects were of Caucasian ethnicity by self-assignment. The study followed the Declaration of Helsinki and was approved by the Coordinating Ethics Committee of the Helsinki University Hospital. Each participating subject provided informed written consent including into the study of pain-relevant genes.

For the present method-establishment and validation, a genetic association analysis with pain status was not intended. Nevertheless, in order to obtain a representative cohort for the evaluation of the frequency of genetic variants, which is as close as possible to a random sample of test subjects, the relative proportion of patients with persistent pain and without persistent pain, as observed in the entire cohort of 1000 women [94], was retained in the composition of the samples. Specifically, for assay establishment, 60 samples were planned. In the above-mentioned cohort, a total of 853 individuals were analyzed, 779 of whom had a favorable outcome with respect to pain, while 74 had developed persistent pain according to criteria defined in [94]. In 60 samples, this ratio corresponds to 5.7 individuals with persistent pain. After rounding, the sample currently analyzed consisted of 55 subjects without pain and six patients with persistent pain.

#### 4.2.2. DNA Amplification

A multiplex PCR amplification strategy for the sequences of the coding genes was accomplished online (Ion Ampliseq^TM^ Designer; http://www.ampliseq.com) to amplify the target region specified above with 25 base pair exon padding. After a comparison of several primer design options, the design providing the maximum target sequence coverage was chosen. The 403kb target-sized panel has been ordered with 1504 amplicons and covered approximately 97.54% of the target sequence. A total of 10 ng DNA per sample was used for the target enrichment by a multiplex PCR and each DNA pool was amplified with the Ion Ampliseq^TM^ Library Kit in conjunction with the Ion Ampliseq^TM^ “custom Primer Pool”-protocols according to the manufacturer procedures (Life Technologies, Darmstadt, Germany).

After each pool had undergone 17 PCR cycles, the PCR primers were removed with FuPa Reagent (Thermo Fisher Scientific, Dreieich, Germany) and the amplicons were ligated to the sequencing adaptors with short stretches of index sequences (barcodes) that enabled sample multiplexing for subsequent steps (Ion Xpress™ Barcode Adapters Kit; Life Technologies, Carlsbad, CA, USA). After purification with AMPure XP beads (Beckman Coulter, Krefeld, Germany), the barcoded libraries were quantified with a Qubit^®^ 2.0 Fluorimeter (Life Technologies, Darmstadt, Germany) and normalized for DNA concentration to a final concentration of 20 pmol/L using the Ion Library Equalizer™ Kit (Life Technologies, Darmstadt, Germany). Equalized barcoded libraries from 9–10 samples at a time were pooled. To clonally amplify the library DNA onto the Ion Sphere Particles (ISPs; Life Technologies, Darmstadt, Germany), the library pool was subjected to emulsion PCR by using an Ion PGM HI-Q View Template Kit on an PGM OneTouch system (Life Technologies, Darmstadt, Germany) following the manufacturer’s protocol.

#### 4.2.3. DNA Sequencing

Enriched ISPs which carried many copies of the same DNA fragment were subjected to sequencing on an Ion 318 Chip to sequence-pooled libraries with 9 to 10 samples. The number of combined libraries that can be accommodated in a single sequencing run depends on the size of the chip, the balance of barcoded library concentration, and the coverage required. The high-capacity 318 chip was chosen (instead of the low-capacity 314 or the medium-capacity 316 chip) to obtain a high sequencing depth of coverage of minimum 30x. Sequencing was performed using the sequencing kit (Ion PGM Hi-Q Sequencing Kit; Life Technologies, Darmstadt, Germany) as per the manufacturer’s instructions with the 200 bp single-end run configuration. This kit contained the most advanced sequencing chemistry available to users of the Ion PGM System (Life Technologies, Darmstadt, Germany).

#### 4.2.4. Assay Validation

The current Ampliseq^TM^ panel is technically identical to the panel which established previously [20], which had been validated by external Sanger sequencing. Hence, no divergences in the current panel were expected. Again, for method validation a genomic region from the *COMT* gene which has already been in focus in a previous study [196], was chosen for validation by Sanger sequencing [197,198] in an independent external laboratory (Eurofins Genomics, Ebersberg, Germany), which was performed in ten DNA samples randomly chosen from the *n* = 72 samples in the present cohort. Amplification of the respective DNA segments was done using PCR primer pairs (forward, reverse) of (i) 5′-CCTTATCGGCTGGAACGAGTT-3′, 5′-GTAAGGGCTTTGATGCCTGGT-3′ (ii) 5′-GTTATTCCTCTGTAAGCAGCTGCCT-3′, 5′-TGTTTGTTTTAGATTGTGGTGGGTT-3′ (iii) 5′-TTTATTGCACAGACTTGCGGGTTC-3′, 5′-AGCCTTTTGAGAGATTTGAGTTTCA-3′.The results of Sanger sequencing were aligned with the genomic sequence and analyzed using Chromas Lite^®^ (Version 2.1.1, Technelysium Pty Ltd., South Brisbane, Australia) and the GenomeBrowse^®^ (Version 2.0.4, Golden Helix, Bozeman, MT, USA) was used to compare the sequences obtained with NGS or Sanger techniques. 

### 4.3. Data Analysis

#### 4.3.1. Bioinformatics Generation of Sequence Information

The raw data (unmapped BAM-files) from the sequencing runs were processed using Torrent Suite Software (Version 5.2.2, Life Technologies, Darmstadt, Germany) to generate read alignments which are filtered by the software into mapped BAM-files using the reference genomic sequence (hg19) of target genes. Variant calling was performed with the Torrent Variant Caller Plugin using as key parameters: minimum allele frequency = 0.15, minimum quality = 10, minimum coverage = 20 and minimum coverage on either strand = 3. The annotation of called variants was done using the Ion Reporter Software (Version 4.4; Life Technologies, Darmstadt, Germany) for the VCF files that contained the nucleotide reads and the SNP & Variation Suite^®^ (SVS) software (Version 8.9.0 for Linux, Golden Helix, Bozeman, MT, USA) to map the sequences to the reference sequences GRCh37 hg19 (dated February 2009). The SNP and Variation Suite software (SVS Version 8.4.4; Golden Helix, Bozeman, MT, USA) was used for the analysis of sequence quality and coverage.

#### 4.3.2. Descriptive Analysis of Variant Frequencies

Variants were identified and assigned to coding, regulatory, intronic or other locations on the genes using the SVS software. Based on the observed allelic frequency, the expected number of homozygous and heterozygous carriers of the respective SNP (single nucleotide polymorphism) according to the Hardy-Weinberg equilibrium was compared with the observed number using Fisher’s exact test [199] as proposed previously [200]. Only variants within the Hardy–Weinberg equilibrium were retained. The number of variants detected was analyzed for correlation with gene length, i.e., the number of nucleotides read for each gene in the present assays. A robust correlation analysis was performed by calculating the percentage bend correlation coefficient using the R-package “WRS2” (https://cran.r-project.org/package=WRS2 [201]). Since the introns were considered only at their edges and intergenic regions only when the primer localization suggested by the panel design software included them, the correlation analysis was repeated for the exonic variants only. In order to recheck the correspondence of the read number of nucleotides with independent information about the length of the respective genes, the latter was retrieved from the Bioconductor Annotation Data Package “org.Hs.eg.db” (https://bioconductor.org/packages/release/data/annotation/html/org.Hs.eg.db.html [202]) using the R library “EDASeq” (https://bioconductor.org/packages/release/bioc/html/EDASeq.html [203]).

#### 4.3.3. Identification of the Functional Genomics Biological Roles of the Set of Pain Genes

The approach to the functional genomics biological roles of the set of pain genes was the same as before for the complete set of pain genes [8,17], or for other contextually selected sets of genes relevant to pain [115]. The methods were described in detail in special publications [17,98]. The biological roles of the set of the *n* = 72 genes of the present panel, versus the biological roles of all human genes, were retrieved via analyses of the Gene Ontology knowledge base (GO; http://www.geneontology.org/) [204,205]. In the GO, knowledge of the biological processes, molecular functions and cellular components of genes is formulated using a controlled and clearly defined vocabulary of GO terms annotated to the genes [8,98]. Here, the biological processes were used to compare the results with previous reports that had used this GO category [8,98]. In the GO, the terms are related by “is-a”, “part-of” and “regulated” relationships and form a polyhierarchy organized in a directed acyclic graph (DAG [97]), with a top-down polyhierarchy starting with more general root terms and specializing in the leaves representing GO terms of narrowest definitions.

In order to obtain the DAG describing the biological processes in which the 72 selected genes are involved, an overrepresentation analysis (ORA) was performed, which compared the occurrence of the specific set of genes annotated to certain GO terms with the expected occurrence of all human genes to these terms. The significance of a GO term associated with the present set of genes was determined using Fisher’s exact tests [199] with a *p*-value threshold of t_p_ < 5 × 10^−15^ and an α correction for multiple testing according to Bonferroni [206]. The conservative thresholds were chosen heuristically, with the criterion that the number of significant GO terms should not exceed the size of the gene set. The analyses were performed using our R library “dbtORA” (https://github.com/IME-TMP-FFM/dbtORA [100]) on the R software environment (version 4.0.2 for Linux, country; http://CRAN.R-project.org/ [92]).

In order to obtain an understandable interpretation of the GO-based functional genomics of pain covered by the selected NGS panel of 72 genes, the information was further reduced. As a basis for the selection of the most appropriate terms to describe the DAG, i.e., the terms that can serve as headlines for each branch of the DAG, the remarkableness measure was previously introduced [98]. That is, for each term $T_{i}$ in the set of terms, its remarkableness, $Rem\left(T_{i}\right)$, was calculated as the product of certainty and information value, i.e., $Rem\left(T_{i}\right)=Cert\left(T_{i}\right)\cdot Info\left(T_{i}\right)$. There, the certainty of a term $T_{i}$ in the significant term set resulting from the ORA, is defined as *Cert*(*T_i_*) = *p* (there is a Term with smaller *p*-value) $=\left|\left\{T_{k}:p-\mathrm{value}\left(T_{k}\right)<p-\mathrm{value}\left(T_{i}\right)\right\}\right|/n_{T}$, where $n_{T}$ denotes the number of significant GO terms annotated to the given set of genes. This reflects how safe it is to assume that the term $T_{i}$ describes the gene set, with numerical values in the interval (0,1). The information value of the term $T_{i}$ can be captured using the (partial) Shannon information calculated as $Info\left(T_{i}\right)=-e\cdot p_{i}\cdot ln\left(p_{i}\right)$ with $p_{i}=n_{G}\left(T_{i}\right)/n_{G}$, where $n_{G}\left(T_{i}\right)$ is the number of genes in the input set annotated to term $T_{i}$ and $n_{G}$ is the number of all genes in the set. By using the factor e and the natural logarithm the values of the information are normalized to the interval (0,1).

In each branch of the DAG, the most remarkable term qualified as a potential heading, i.e., GO terms that succinctly summarize the biological processes covered by the branch of the polyhierarchy in which they represent the most remarkable term. Of the GO terms that lend themselves to being headlines, the most important subset was identified using an item categorization technique implemented as computed ABC analysis, which meets the basic requirements of feature selection using filtering techniques [207]. ABC analysis aims to divide a data set into three disjoint subsets named “A”, “B”, and “C”. Set “A” should contain the “important few” elements, i.e., those elements that make it possible to achieve maximum yield with minimum effort [208]. Sets “B” and “C” include elements where an increase in expenditure is proportional to an increase in yield or the “trivial many”, respectively. Hence, GO terms that were members of ABC set “A” were retained as most significant to the functional genomics covered by the 72 genes in the present NGS panel. The calculations were performed using the R package “ABCanalysis” (http://cran.r-project.org/package=ABCanalysis [99]). As this provided still many GO terms, a more global abstraction was obtained by applying the method of “subsumption” as introduced previously in the context of functional abstraction as a method to discover knowledge in gene ontologies [98]. That is, let *T* be a term in a specific ontology which covers the terms *T_1_*,…,*T_k_*. A set of headlines *H* containing *T_1_*,…,*T_k_* is abstracted if *T_1_*,…,*T_k_* are replaced by *T* in *H*, thereby reducing the number of headlines. This aimed for a 5–9 final terms describing the polyhierarchy, which would ideally be within Miller’s optimum of a human understandable size of a set of objects [209].

## 5. Conclusions

A 72-pain gene NGS panel is proposed that covers (i) a subset of 29 genes identified previously in a bioinformatics approach as key genes covering the biological functions of 540 genes relevant to pain by 70% [17]. Additional genes that were included, had been independently proposed as relevant for persistent pain [9,10], and the functional focus of the whole panel was now on immune or inflammatory processes, in line with the increasing evidence that such processes are key players in persistent pain [115]. Together with a recently established AmpliSeq™ panel of 77 further pain-relevant genes [20], the assay covers a relevant part of the current state of knowledge on the genetic architecture of persistent pain (Figure 1). The assay is applicable for small to large-scale experimental setups to access information about any nucleotide within the coding and regulatory portions of pain-relevant genes in a study cohort at costs per raw megabase which are in line with contemporary genotyping costs across different technical methods of NGS. In the genotypes of the 61 subjects studied in the context of the present assay establishment, tens of variants were found that had previously been reported with functional implications for pain, pharmacogenetics of analgesics, and for pharmacological treatments not related to pain.

## Figures and Tables

**Table 1:** Overview of the *n* = 72 genes contained in the proposed NGS panel. Subset 1 includes d = 29 genes identified using a computational functional genomics-based approach in which the gene sets are reduced to the most relevant items based on the importance of the gene within the polyhierarchy of biological processes characterizing the disease [17]. Subset 2 resulted from the intersection of two proposed sets of human genes involved in modulating the risk or clinical course of persistent pain “Mogil” [9], and “Zorina-Lichtenwalter”.

Gene Symbol	NCBI	Gene Description	Reference
Subset #1			
*ADRA2A*	150	Adrenoceptor alpha 2A	[22]
ADRB2 *	154	Adrenoceptor beta 2	[23]
*AGER*	177	Advanced glycosylation end-product specific receptor	[24]
APOE *	348	Apolipoprotein E	[25]
*CCL21*	6366	C-C motif chemokine ligand 21	[26]
*CCL5*	6352	C-C motif chemokine ligand 5	[27]
*CCR2*	729230	C-C motif chemokine receptor 2	[28]
*CCR7*	1236	C-C motif chemokine receptor 7	[29]
*CD4*	920	CD4 molecule	[30]
*CD40*	958	CD40 molecule	[31]
*CD74*	972	CD74 molecule	[32]
*CHRNA7*	1139	Cholinergic receptor nicotinic alpha 7 subunit	[33]
*DRD1*	1812	Dopamine receptor D1	[34]
DRD2 *	1813	Dopamine receptor D2	[34]
*EDN1*	1906	Endothelin 1	[35]
*F2R*	2149	Coagulation Factor II thrombin receptor	[36]
*F2RL1*	2150	F2R like trypsin Receptor 1	[36]
*IFNG*	3458	Interferon gamma	[37]
IL1B *	3553	Interleukin 1 beta	[38]
*IL6*	3569	Interleukin 6	[39]
*LYN*	4067	LYN proto-oncogene, Src family tyrosine kinase	[40]
*MAPK1*	5594	Mitogen-activated protein kinase 1	[41]
OPRM1 *	4988	Opioid receptor mu 1	[42]
P2RX7 *	5027	Purinergic receptor P2X 7	[43]
*PRKCA*	5578	Protein kinase C alpha	[44]
*PRKCD*	5580	Protein kinase C delta	[45]
*TLR4*	7099	Toll-like receptor 4	[46]
*TLR9*	54106	Toll-like receptor 9	[47]
TNF*	7124	Tumor necrosis factor	[48]
Subset #2			
*ACAN*	176	Aggrecan	[49]
*ACE*	1636	Angiotensin I converting enzyme	[50]
*ADRA1D*	146	Adrenoceptor alpha 1D	[51]
*ADRA2C*	152	Adrenoceptor alpha 2C	[52]
ADRB2 *	154	Adrenoceptor beta 2	[53]
APOE *	348	Apolipoprotein E	[25]
*AR*	367	Androgen receptor	[54]
*CALCA*	796	Calcitonin related polypeptide alpha	[55]
*CASP9*	842	Caspase 9	[56]
*CFTR*	1080	CF transmembrane conductance regulator	[57]
*CRHBP*	1393	Corticotropin releasing hormone binding protein	[58]
*COMT*	1312	Catechol-O-methyltransferase	[59]
DRD2 *	1813	Dopamine receptor D2	[34]
*DRD4*	1815	Dopamine receptor D4	[60]
*ESR1*	2099	Estrogen receptor 1	[61]
*GCH1*	2643	GTP cyclohydrolase 1	[62]
*GDF5*	8200	Growth differentiation factor 5	[63]
*GSTM1*	2944	Glutathione S-transferase mu 1	[64]
*HLA-DRB1*	3123	Major histocompatibility complex, class II, DR beta 1	[65]
*HTR2A*	3356	5-hydroxytryptamine receptor 2A	[66]
*IL1A*	3552	Interleukin 1 alpha	[67]
*IL10*	3586	Interleukin 10	[68]
IL1B *	3553	Interleukin 1 beta	[38]
*IL1RN*	3557	Interleukin 1 receptor antagonist	[69]
*CXCL8*	3576	C-X-C motif chemokine ligand 8	[70]
*KCNS1*	3787	Potassium voltage-gated channel modifier subfamily S member 1	[71]
*MAOA*	4128	Monoamine oxidase A	[72]
*MC2R*	4158	Melanocortin 2 receptor	[73]
*MTHFD1*	4522	Methylenetetrahydrofolate dehydrogenase, cyclohydrolase and formyltetrahydrofolate synthetase 1	[74]
*MTRR*	4552	5-methyltetrahydrofolate-homocysteine methyltransferase reductase	[75]
*NFKBIA*	4792	NFKB inhibitor alpha	[76]
*NR3C1*	2908	Nuclear receptor subfamily 3 group C member 1	[77]
OPRM1 *	4988	Opioid receptor mu 1	[42]
P2RX7 *	5027	Purinergic receptor P2X 7	[43]
*PGR*	5241	Progesterone receptor	[78]
*POMC*	5443	Proopiomelanocortin	[79]
*PRSS1*	5644	Serine protease 1	[80]
*PTGS2*	5743	Prostaglandin-endoperoxide synthase 2	[81]
*SCN9A*	6335	Sodium voltage-gated channel alpha subunit 9	[82]
*SERPINA6*	866	Serpin family A member 6	[83]
*SHMT1*	6470	Serine hydroxy methyltransferase 1	[84]
*SMAD3*	4088	SMAD family member 3	[85]
*SOD2*	6648	Superoxide dismutase 2	[86]
*SPINK1*	6690	Serine peptidase inhibitor, Kazal type 1	[57]
*STAT6*	6778	Signal transducer and activator of transcription 6	[87]
*TGFB1*	7040	Transforming growth factor beta 1	[88]
TNF *	7124	Tumor necrosis factor	[48]
*TRPA1*	8989	Transient receptor potential cation channel subfamily A member 1	[89]
*TRPM8*	79054	Transient receptor potential cation channel subfamily M member 8	[89]
*TRPV1*	7442	Transient receptor potential cation channel subfamily V member 1	[89]

*: Gene occurs in both subsets.

**Table 2:** Variants with reported clinical effects. The table provides a list of human variants in the 72 putative chronic pain genes which were found in the present random sample of 61 subjects of Caucasian ethnicity, for which clinical associations have been reported.

Gene Name	Variant	DbSNP ^#^ Accession Number	Consequence	Known Clinical Association	Reference
Pain Related					
*ACAN*	chr15:g.89398553C>A	rs35430524	NON_SYNONYMOUS	Chronic low back pain	[49]
*ACAN*	chr15:g.89402051A>G	rs1042630	NON_SYNONYMOUS	Chronic low back pain	[49]
*ADRB2*	chr5:g.142780339C>A	rs1042718	NON_SYNONYMOUS	Associated with increased response to fentanyl	[118]
*ADRB2*	chr5:g.148206917C>T	rs1042719	SYNONYMOUS	Associated with chronic pain in sickle cell disease	[119]
*ADRB2*	chr5:g.148207447G>A	rs1042720	SYNONYMOUS	Associated with chronic pain in sickle cell disease	[119]
*COMT*	chr22:g.19951207C>T	rs4818	SYNONYMOUS	Chronic post-surgical pain	[120]
*ESR1*	chr6:g.152420095G>A	rs2228480	SYNONYMOUS	Risk of knee osteoarthritis	[121]
*HTR2A*	chr13:g.47409034G>A	rs6314	NON_SYNONYMOUS	Development of rheumatoid arthritis	[122]
*IL1RN*	chr2:g.113887207T>C	rs419598	SYNONYMOUS	Altered pain perception	[123]
				Knee osteoarthritis	[124]
*IL1RN*	chr2:g.113890304T>C	rs315952	SYNONYMOUS	Osteoarthritis	[125]
*IL6*	chr7:g.22771039T>C	rs13306435	SYNONYMOUS	Associated with persistent lumbar radicular pain	[126]
*KCNS1*	chr20:g.43723627T>C	rs734784	NON_SYNONYMOUS	Pain variability	[127]
*MTRR*	chr5:g.7885959A>G	rs162036	NON_SYNONYMOUS	Associated with migraine	[128]
*P2RX7*	chr12:g.121592689T>C	rs17525809	NON_SYNONYMOUS	Regulate the onset of gouty arthritis	[129]
*P2RX7*	chr12:g.121600253T>C	rs208294	NON_SYNONYMOUS	Pain tolerance	[130]
				Variability in chronic pain sensitivity	[43]
*P2RX7*	chr12:g.121615103G>A	rs1718119	NON_SYNONYMOUS	Cold pain sensitivity and analgesic effect of fentanyl	[131]
*P2RX7*	chr12:g.121622304A>G	rs3751143	NON_SYNONYMOUS	Relevance to diabetic neuropathic pain	[132]
*SCN9A*	chr2:g.167141109G>C	rs41268673	NON_SYNONYMOUS	Oxaliplatin induced neuropathy	[133]
	chr2:g.166277030T>G	rs12478318	NON_SYNONYMOUS	Genotype GT is associated with Pain Insensitivity, Congenital as compared to genotype TT	[134]
*TGFB1*	chr19:g.41858876C>T	rs1800471	NON_SYNONYMOUS	Painful bladder syndrome	[135]
				Insensitivity to pain and erythromelalgia	[136]
*TRPA1*	chr8:g.72975801T>C	rs7819749	SYNONYMOUS	Sensitivity to heat stimuli and topically applied capsaicin	[16]
*TRPM8*	chr2:g.234854550G>A	rs11562975	SYNONYMOUS	Attenuated cold pain sensation	[137]
*TRPV1*	chr17:g.3494361G>T	rs222748	NON_SYNONYMOUS	Burning pain and capsaicin sensitivity	[138]
*TRPV1*	chr17:g.3494361G>T	rs222748	NON_SYNONYMOUS	Burning pain and capsaicin sensitivity	[138]
				Cold and heat pain sensitivity	[139]
*TRPV1*	chr17:g.3495374G>A	rs222749	NON_SYNONYMOUS	Chronic migraine	[55]
				Altered pain perception	[140]
Non-Pain Related					
ACAN	chr15:g.89398407C>T	rs3743398	NON_SYNONYMOUS	Glioblastoma multiforme	[141]
ACE	chr17:g.61564052A>G	rs4331	SYNONYMOUS	Risk of late-onset Alzheimer’s disease	[142]
CASP9	chr1:g.15833506C>T	rs2308950	NON_SYNONYMOUS	Risk of non-Hodgkin’s lymphoma	[143]
				Predisposition to lung cancer	[144]
CASP9	chr1:g.15834360A>G	rs2020902	SPLICE_SITE	Bladder cancer risk	[145]
CCR2	chr3:g.46399174G>T	rs3918367	SYNONYMOUS	Associated with endothelial function in prediabetic individuals	[146]
CCR2	chr3:g.46399208G>A	rs1799864	NON_SYNONYMOUS	Associated with prostatic hyperplasia and prostate cancer	[147]
CCR2	chr3:g.46399798T>C	rs1799865	SYNONYMOUS	Associated with markers of exercise-induced skeletal muscle damage	[148]
CD4	chr12:g.6924109C>T	rs11575099	SYNONYMOUS	Involved in multiple sclerosis	[149]
				Stressful life events and suicide	[150]
CFTR	chr7:g.117175372A>G	rs121909046	NON_SYNONYMOUS	Associated with respiratory and pancreatic diseases	[151]
CFTR	chr7:g.117199709G>C	rs1800095	NON_SYNONYMOUS	Associated with idiopathic pancreatitis	[152]
CFTR	chr7:g.117235055T>A	rs1042077	SYNONYMOUS	Associated with cystic fibrosis	[153]
ESR1	chr6:g.152129308G>A	rs746432	SYNONYMOUS	Breast cancer risk	[154]
HTR2A	chr13:g.47409034G>A	rs6314	NON_SYNONYMOUS	Major depressive disorder	[155]
HTR2A	chr13:g.47409149T>A	rs35224115	SYNONYMOUS	Obsessive-compulsive disorder	[156]
HTR2A	chr13:g.47466622G>A	rs6305	SYNONYMOUS	Schizophrenia	[157]
IL1RN	chr2:g.113877713A>C	rs878972	SPLICE_SITE	Prostate cancer risk	[158]
IL6	chr7:g.22771156C>G	rs2069849	NON_SYNONYMOUS	Associated with obesity	[159]
MTRR	chr5:g.7878424T>A	rs2303080	NON_SYNONYMOUS	Risks of spina bifida and conotruncal heart defects	[160]
MTRR	chr5:g.7889216G>A	rs2287779	SYNONYMOUS	Risk of childhood acute lymphoblastic leukemia	[161]
MTRR	chr5:g.7889304C>T	rs2287780	NON_SYNONYMOUS	Gastric cancer risk	[162]
NR3C1	chr5:g.142661490A>G	rs6196	SYNONYMOUS	Associated with corticosteroid dependency and resistance	[163]
NR3C1	chr5:g.142662280G>T	rs258751	NON_SYNONYMOUS	Associated with high-altitude pulmonary edema	[164]
NR3C1	chr5:g.142779317T>A	rs56149945	NON_SYNONYMOUS	Associated with cocaine use	[165]
NR3C1	chr5:g.142780337C>G	rs6190	NON_SYNONYMOUS	Associated with mood and anxiety disorders in patients with asthma	[166]
NR3C1	chr5:g.142780339C>A	rs6189	NON_SYNONYMOUS	Associated with mood and anxiety disorders in patients with asthma	[166]
P2RX7	chr12:g.121600238G>T	rs28360447	STOP_GAINED	Osteoporosis risk	[167]
PGR	chr11:g.100909991T>C	rs500760	SYNONYMOUS	Gastric cancer risk	[168]
PGR	chr11:g.100922202G>A	rs1042839	SYNONYMOUS	Sporadic neuroendocrine tumor risk	[169]
PRKCA	chr17:g.64685078G>A	rs2227857	SYNONYMOUS	Deep vein thrombosis	[170]
POMC	chr2:g.25387624G>A	rs8192605	SYNONYMOUS	Associated with substance dependence and body mass index	[171]
SERPINA6	chr14:g.94772504G>A	rs1042394	SYNONYMOUS	Associated with stress fractures	[172]
SERPINA6	chr14:g.94776221A>C	rs2228541	NON_SYNONYMOUS	Lymphoblastic leukemia	[173]
TLR4	chr9:g.120475302A>T	rs4986790	NON_SYNONYMOUS	Higher risk for gastric cancer	[174]
	chr9:g.120475602C>T	rs4986791	NON_SYNONYMOUS	Associated with lower respiratory tract infections	[175]
TRPM8	chr2:g.234905078C>A	rs11563208	SYNONYMOUS	Associated with cold-induced airway hyperresponsiveness in bronchial asthma	[176]
TRPV1	chr17:g.3495391T>C	rs55916885	NON_SYNONYMOUS	Associated with asthma	[177]

^#^https://www.ncbi.nlm.nih.gov/snp/?cmd=search.

**Table 3:** Gene variants with reported pharmacogenetic effects. The table provides a summary of variants in genes included in the proposed panel of *n* = 72 genes and found in the DNA of the 61 analyzed subjects, that have been implicated in a pharmacogenetic context to modulate the effects of drugs administered for the treatment of pain or as disease modifying therapeutics in a painful disease.

Gene Name	Variant	Affected Drug	Findings	Reference
*ADRA2A*	rs1800545	Oxycodone	Allele A is associated with dose of opioids in people with Pain as compared to allele G in the development sample	[178]
*ADRA2A*	rs11195419	Oxycodone	Allele A is associated with dose of opioids in people with Pain as compared to allele G in the development sample	[178]
*ADRB2*	rs1042718	Fentanyl	Genotype AC is associated with increased response to fentanyl in healthy individuals as compared to genotype CC	[118]
*CALCA*	rs3781719	Botulinum	Patients with the AA genotype and chronic migraine may have an increased response to botulinum toxin A as compared to patients with the AG or GG genotypes.	[55]
*CCL21*	rs2812378	Infliximab	Nominal association of this SNP with association of rheumatoid arthritis risk alleles	[179]
*CD40*	rs1126535	Adalimumab	Allele T is associated with increased response to adalimumab in people with Arthritis	[180]
*COMT*	rs4633	Morphine	Patients with the CC genotype may be more likely to require postoperative intervention with opioids after adenotonsillectomy as compared to patients with the TT genotype. Other genetic and clinical factors may also influence a patient’s requirement for pain management.	[181]
*DRD2*	rs6275	Heroine	Polymorphism is associated with decreased likelihood of headache disorders	[182]
*ESR1*	rs9340799	Leflunomide	Patients with the AA genotype may experience greater response to leflunomide as compared to patients with the GG genotype. Other genetic and clinical factors may also influence response to leflunomide, particularly rs2234693.	[183]
*IFNG*	rs2069705	Etanercept	Allele G is associated with increased response to Tumor necrosis factor alpha (TNF-alpha) inhibitors in people with Arthritis	[184]
*IL1B*	rs1143634	Morphine	Allele A is associated with increased dose of morphine in women with Pain	[185]
*IL6*	rs11265618	Tocilizumab	Patients with the CC genotype and rheumatoid arthritis may have a better response when treated with tocilizumab as compared to patients with the CT or TT genotype	[186]
*MTRR*	rs1801394	Folic Acid	Female patients with the AA genotype and Migraine who are treated with folic acid and a vitamin b-complex may have decreased severity of pain as compared to patients with the GG genotype.	[187]
*OPRM1*	rs1799971	Opioids	Allele G is associated with increased plasma concentrations of morphine in women with Pain, Postoperative as compared to allele A	[188]
*P2RX7*	rs1718125	Fentanyl	Patients with the CC genotype may have decreased fentanyl dosage requirements as compared to patients with the CT or TT genotypes	[131]
*PTGS2*	rs20417	Ibuprofen	Patients with the CC genotype may have decreased pain relief to ibuprofen as compared to patients with GG or CG genotype.	[189]; however, see [190]

## Data Availability

The original data containing the patients’ genetic information cannot be shared due to data security restrictions.
